# Technical Note and Short Review: A Challenging Case of a Premature Newborn Presenting With ICH, IVH, Ventriculitis, and Hydrocephalus

**DOI:** 10.1002/ccr3.70990

**Published:** 2025-10-03

**Authors:** Hannes Egermann, Christian Knorr, Basheer Al‐Shameri, Adolf Müller, Markus Denzinger, Stephan Lackermair

**Affiliations:** ^1^ Department of Neurosurgery Krankenhaus Barmherzige Brüder Regensburg Germany; ^2^ Department of Pediatric Surgery and Pediatric Orthopedics University Children's Hospital Regensburg (KUNO) Regensburg Germany

**Keywords:** hydrocephalus, ICH, IVH, ventriculitis, ventriculo peritoneal shunt, ventriculosopy

## Abstract

Premature infants are at a heightened risk of intracerebral or intraventricular hemorrhages (IVH), which often lead to posthaemorrhagic hydrocephalus and necessitate the placement of a ventriculoperitoneal (VP) shunt. Here, we present a case involving suspected ICH, which led to multiloculated hydrocephalus, recurrent ventriculitis, and meningitis. This case study highlights the complexities of managing infections and the importance of endoscopic surgery in restoring CSF flow. It also sheds light on the long‐term developmental outlook when treating premature infants with multilocular hydrocephalus.

## 1 Introduction/Object

Premature infants are at significantly increased risk of spontaneous intracerebral hemorrhages, which are often associated with ventricular system involvement and posthaemorrhagic hydrocephalus [1]. In such cases, temporary cerebrospinal fluid drainage is often necessary, either via repeated lumbar punctures or the placement of a lumbar drain or external ventricular drain [2]. This phase of early childhood development is also susceptible to infection, which can lead to repeated invasive procedures, including potentially complicated ventriculoperitoneal shunt placements [2, 3, 4]. We present the case of a 19‐month‐old male infant who experienced the aforementioned cascade as a premature infant and was acutely admitted to hospital with an unclear infectious condition suggestive of ventriculitis or meningitis. This report aims to shed light on intracerebral hemorrhages in early childhood, the occurrence of posthaemorrhagic, often multiloculated, hydrocephalus and associated post‐interventional infections. Additionally, we describe an individualized endoscopic ventriculoscopy approach for managing this condition.

## 2 Report/Method

We were introduced to a 19‐month‐old child who was born prematurely. The child was delivered at 32 weeks and 0 days of gestation. We do not have specific details from external sources. However, retrospectively, the prematurity at 32 weeks of gestation is the most significant risk factor. To our knowledge, there were no indications of a high‐risk pregnancy or infection. The mother was also not a primipara. Postpartum, the infant exhibited significantly reduced adaptation responses. Although the Apgar score is not applicable to preterm infants (< 37 weeks of gestation), it was clearly documented to be below four points [5]. Consequently, the suspicion of a spontaneous intracerebral hemorrhage was obvious. The early ultrasound examination revealed a suspected formation in the ventricular system, which at that time could not be identified with certainty as blood or possibly an infection. CSF obtained indicated the presence of an infection (CSF cell count 3296/μL, protein 757 mg/dL, glucose 5 mg/dL, lactate 6.4 mmol/L; multiplex meningitis PCR: negative). A later examination showed a “massive” periventricular hemorrhage with pronounced haemorrhagic infarction. Infection and significant intracranial hemorrhage were therefore two significant risk factors in this case for the development of hydrocephalus [2, 6].

The child experienced a problematic cascade of multiloculated hydrocephalus, requiring repeated CSF punctures, either lumbar or via the placement of ventricular catheters. Recurrent ventriculitis and meningitis further complicated matters. Microbiological analysis of the CSF detected *Acinetobacter ursingii* , Micrococcus sp., Corynebacterium pseudodiphtheria, and *Staphylococcus epidermidis* in the CSF. The interpretation was challenging, and the findings were mostly considered contamination. Approximately six procedures were necessary to place Rickham catheters and various ventricular catheters. At the extern hospital, four VP shunt replacements and an additional four revisions of the VP shunts or their implant components were required. This resulted in two ventricular catheters being retained in the left lateral ventricle, presumably due to dislocation during revision surgery.

At the time of the initial presentation to our institution, the developmentally delayed child presented with fever, vomiting, and other abdominal symptoms, such as tenderness, flatulence, and loss of appetite. Neurologically, the child appeared alert. It offered gaze fixation and moved all extremities laterally equally. The pupils were isocoric, with equal direct and indirect light responses. Laboratory and clinical findings indicated possible appendicitis. This was confirmed by abdominal sonography and an abdominal MRI scan. Therefore, laparoscopy was performed to treat the condition. However, during the procedure, catheter‐associated peritonitis was observed, with a peritoneal shunt component located in the right lower abdomen. A CSF sample revealed the presence of *Staphylococcus epidermidis* . The peritoneal shunt catheter was externalized, and antibiotic therapy with meropenem and vancomycin was initiated. As CSF clearance was not achieved, it was hypothesized that recurrent infections were being caused by retained and possibly colonized remnants of the shunt catheters in the left lateral ventricle and that their surgical removal would be advisable.

**FIGURE 1:**
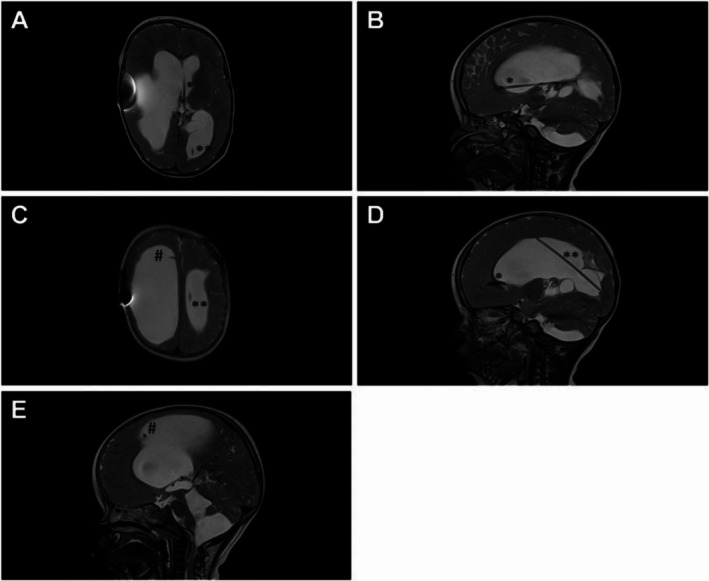
T2 MRI images: A (transversal), B (sagittal) showing frontally the lost catheter on the floor of the left lateral ventricle (*) and in the occipital horn the second lost catheter (**); C (transversal) showing the 2nd lost catheter in the left ventricle (**) as well as on the right frontal side the existing VP shunt catheter (#); additionally, in the sagittal image (D), the lost catheter (*) is shown in the frontal lobe; E (sagittal) enlargement of the 4th ventricle.

**FIGURE 2:**
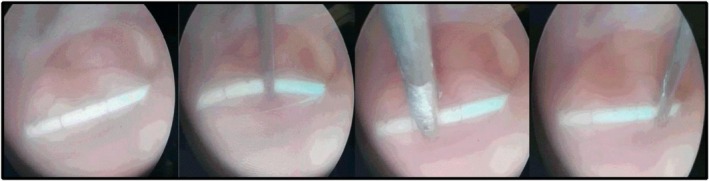
Screenshots from intraoperative endoscopic video of the removal of the catheters.

**FIGURE 3:**
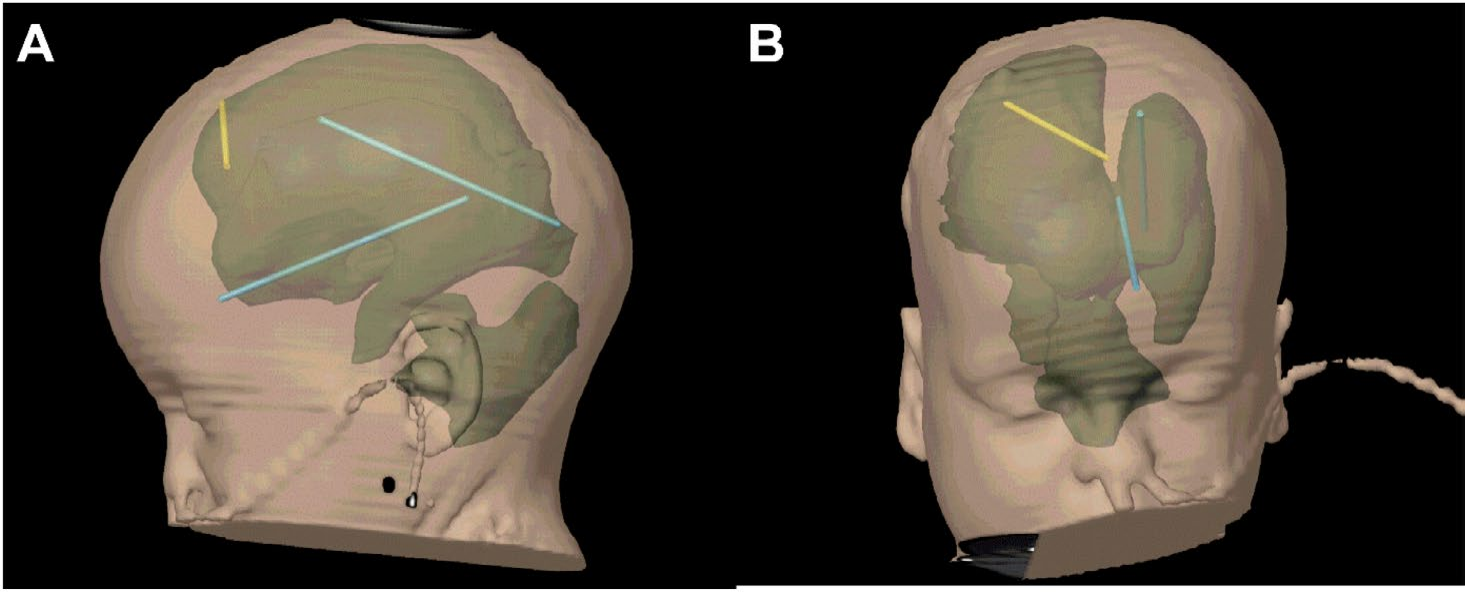
Screenshots of an iplan cranial 3.0 segmentation of the preoperative ventricle volume and the “lost” catheters (blue) as well as the recent VP‐Shunt‐catheter (yellow).

An MRI scan of the skull revealed multiloculated hydrocephalus, characterized by enlargement of the lateral, third, and fourth ventricles, as well as the cisterna magna. This indicated that operative endoscopic restoration of intracranial CSF circulation was necessary (Figure 1). Neither ultrasound nor MRI demonstrated communication between the compartments. Therefore, we decided to study the internal CSF spaces using a contrast medium, administering a radiopaque contrast agent (approximately 2 mL of Solutrast, Bracco Imaging Deutschland GmbH, Konstanz, Germany). A cranial CT scan performed two hours after administration of the contrast agent revealed that the lateral and third ventricles were not communicating with the fourth ventricle or the cisterna magna. In order to assess endoscopic options such as ventriculocisternostomy or catheter balloon dilation of the aqueduct, we planned a two‐stage operative approach involving primarily diagnostic, navigated ventriculoscopy to retrieve any remaining shunt catheter fragments and to inspect the intraventricular conditions individually (Figures 2 and 3). Secondly, we would establish an appropriate CSF diversion, either intracranially or peripherally.

Summarized, we treated the child as follows:

Under initial anesthesia, the ventriculography described above was performed, which showed no communication between the supratentorial and infratentorial cerebrospinal fluid spaces in a known multilocular hydrocephalus with dilatation of the 4th ventricle.

The supratentorial ventricular system was then repaired under the same anesthesia. Navigated with the Brainlab system (iplan cranial 3.0; Curve Platform; Brainlab AG Munich, Germany), the endoscope could be inserted right‐frontally in a favorable trajectory to reach all ventricular compartments after removal of the subcutaneous VP shunt remnant. (Aesculap 0° or 30° optic; Karl Storz, Tuttlingen, Germany) Endoscopy was facilitated by a self‐retaining pneumatic arm of the Unitrac system (Aesculap Unitrac, B. Braun Deutschland GmbH & Co. KG, Tuttlingen, Germany). With an open septum pellucidum, the remaining floating left ventricular catheter could be visualized. As it was not possible to fix the catheter for retrieval via the endoscope trocar, a Yasargil nicola micro forceps (Aesculap FD222R) was used parallel to the trocar. This was used to grasp and remove the catheter that was still fixed frontally and that remained occipitally. Interventional, slight bleeding resolved spontaneously with irrigation and waiting.

The ventricular system was extensively and continuously irrigated. Finally, an external ventricular drain (VentriGuard 8.5 French, Neuromedex GmbH, Hamburg, Germany) was left in place. This was used in the neuropaediatric intensive care unit for 14 days to check the CSF clearance and for intraventricular antibiotic treatment. Staph. epidermidis was detected in the samples. A fungal PCR remained negative (Actinomyces spp. PCR: 16S rDNA). The pathogen was sensitive to vancomycin, linezolid, rifampicin, and fosfomycin. It was resistant to flucloxacillin, cefazolin, and ceftazidime. In our case, we chose vancomycin according to Bayston et al. [3, 7] as the antibiotic agent of choice for treating intraventricularly as well as intravenously.

**FIGURE 4:**
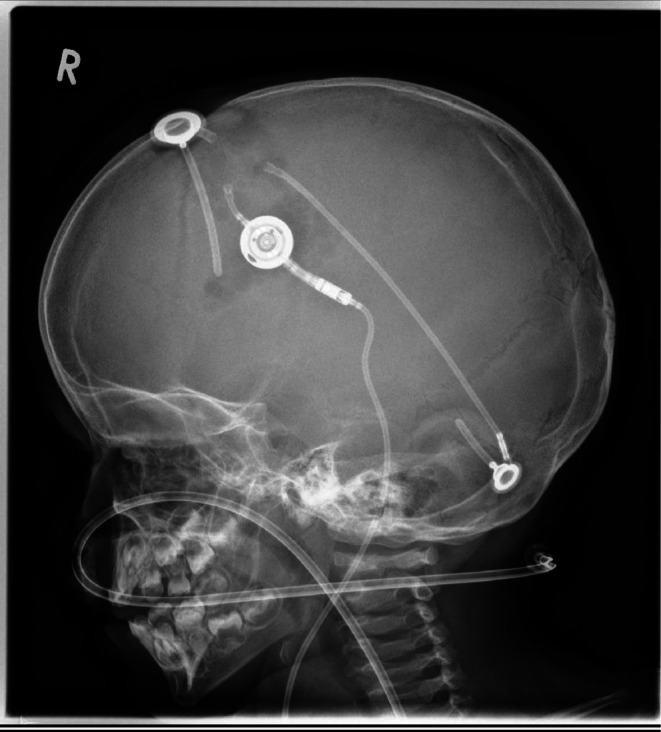
Postoperative X‐ray of the new cranial Shunt‐System established in our institution.

Finally, a new shunt system was implanted in a second operation with CSF clearance under perioperative antibiotic prophylaxis with vancomycin. This was performed on the right frontal side with a ventricular catheter and an additional, ultrasound‐guided suboccipital catheter of the 4th ventricle. The catheters were proximally connected to a Miethke proGAV 2.0 valve (Christoph Miethke GmbH und Co. KG; Potsdam, Germany; proGAV 2.0 valve with SA 2.0, current setting at discharge 13/38 cmH_2_O) using a Y‐shaped connector and finally subcutaneously tunneled and placed intraperitoneally under laparoscopic view (Figure 4). All catheters were chosen to be antibiotic‐coated (antibiotic impregnated catheters with rifampicin and clindamycin; Bactiseal Codman).

**FIGURE 5:**
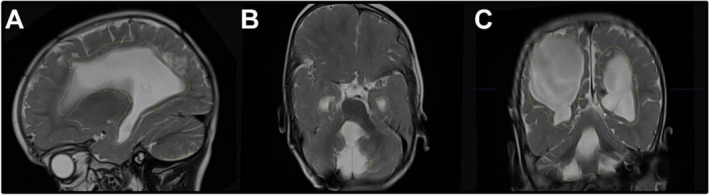
Screenshots of an iplan cranial 3.0 segmentation of the preoperative ventricle volume in comparison to an early control MRI objectifying the reduction of the ventricle size.

The child made a quick recovery. However, due to their global developmental delay, there was no clear neurological improvement in their overall condition during the first six weeks after the operation. As can be seen in Figure 5, the segmentation of ventricular volumes pre‐ and postoperatively shows that they are becoming slightly smaller after three weeks postoperatively. During the 18‐month follow‐up period, no further infections were documented, the child made good progress, and there were no new hospitalizations or operations.

## 3 Discussion

Intracerebral hemorrhage (ICH) in premature infants presents significant clinical challenges, often resulting in complications such as multiloculated hydrocephalus and recurrent ventriculitis. (This discussion will focus on the epidemiology, risk factors, clinical features, investigation, and management strategies associated with these conditions.)

### 3.1 Epidemiology

Intraventricular hemorrhage (ICH) is a common complication in premature infants. There is a significant increase in the incidence of ICH in babies born before 34 weeks of gestation, with an incidence rate of 90%. Furthermore, 40% of premature babies weighing ≤ 1500 g at birth experience an intraventricular hemorrhage [1, 8]. Posthemorrhagic hydrocephalus is a common sequela, occurring in approximately 1%–2% of affected preterm infants. Compared to full‐term babies, the prevalence of hydrocephalus is 2/1000 [9].

### 3.2 Risk Factors

Prematurity itself is a significant risk factor for ICH due to the fragility of the germinal matrix and immature vascular structures. Additional risk factors include birth trauma, hypoxic insults, congenital coagulopathies (e.g., hemophilia), respiratory distress syndrome, prematurity associated with increased venous and arterial blood pressure, and immature cerebral vascular autoregulation or structural abnormalities (e.g., AVMs, aneurysms, tumors, and congenital CNS malformations) [10, 11, 12, 13].

The presence of infections (e.g., meningitis) further exacerbates the risk of developing hydrocephalus. In postmeningitic children, the balance between cerebrospinal fluid production and drainage is disturbed. 31% of children with meningitis develop hydrocephalus malresorptivus. The development of multilocular hydrocephalus is also significantly increased. Mature infants have a risk of 0.13%–0.37% of suffering bacterial meningitis, whereas premature infants have a risk of 1.36%–2.24% [11]. These conditions require careful management due to the complexity of maintaining cerebrospinal fluid (CSF) circulation and preventing infections.

### 3.3 Clinical Features

The clinical presentation of ICH in neonates is often non‐specific, with symptoms ranging from irritability and apnoea to seizures, cyanosis, vomiting, a tense fontanelle, shrill cry, gaze palsy or central facial weakness, and altered consciousness [10]. In cases of hydrocephalus, signs of increased intracranial pressure may be observed, such as a tense fontanel or rapid head growth.

In 1992, Lin et al. described the four phases of brain damage that occur following an intraventricular hemorrhage. The first phase is caused by the hemorrhage itself, as well as the associated infarction and hypoxic insults. The second phase predominantly occurs postnatally due to shock resulting from disturbed cerebral autoregulation, followed by hypotension, increased intracranial pressure, and restricted cerebral perfusion. The third phase occurs when hydrocephalus develops, which is associated with a further increase in ICP. The fourth phase includes shunt blockade, which leads to increased intracranial pressure and, eventually, ventriculitis or isolated fourth ventricle syndrome [12]. However, untreated hydrocephalus certainly leads to developmental delay [1, 14]. There is a risk of blindness, learning difficulties, and even death [6, 15].

### 3.4 Investigation

Diagnosis typically involves neuroimaging techniques. Transfontanel transcranial ultrasound is the gold standard examination for the detection of ICH/IVH in newborns, as it is noninvasive, does not require sedation, and does not involve X‐ray or radiation exposure [8, 10]. Up to 5% of otherwise asymptomatic full‐term newborns may show ICH with ultrasound. The incidence of symptomatic IVH in full‐term neonatal infants is quoted as around 5.2/10,000 [16].

As a further diagnostic test, cMRI is particularly important. Not only does MRI show hemorrhages, it also enables the age of the hemorrhage to be assessed. It can also detect the causes of ICH/IVH, such as the presence of vascular lesions. MRA examinations can nowadays visualize aneurysms smaller than 3 mm [10].

CT scans of premature babies and newborns are also highly sensitive to ICH/IVH. However, this method is not preferred due to the X‐ray radiation involved. Nevertheless, the classical Papile et al. (1978) grading system for intraventricular hemorrhage is based on CT examinations and can also be applied to sonography. Grades 1 and 2 are associated with spontaneous remission, whereas Grades 3 and 4 are associated with the development of progressive hydrocephalus requiring intervention [1, 12, 17].

### 3.5 Management Plan

Managing hydrocephalus and ventriculitis involves a multidisciplinary approach. The conservative treatment approach includes medication. Antidiuretic drugs have been widely used in the treatment of hydrocephalus since the late 1950s. However, Kennedy et al. (2001) proved in their randomized controlled trial of posthaemorrhagic hydrocephalus in infancy, treated with acetazolamide and furosemide, that usage does not reduce the rate of shunt placements [18]. Addressing potential infections with targeted antibiotic therapy is essential in treating and preventing recurrent infections.

Surgical intervention is used to restore CSF circulation and remove obstructions. VP shunt creation is generally associated with risks. Described risk factors include prematurity, being underweight, progressive infections, previous revisions, and altered anatomy, particularly in cases of multilocular hydrocephalus [2, 19].

Lin et al. were able to show better six‐month survival in children weighing more than 3.7 kg compared to children weighing < 2 kg. In addition, a lower CSF protein is prognostically favorable (0.64 g/L vs. 1.39 g/L) [12].

James et al. 1984 and 1987 were able to show that shunt infections are significantly more frequent in the group of premature babies due to the immature immune defense. The risk is estimated at 26.9% for premature babies and only 13% for normal babies [8, 20].

Datas from the Hydrocephalus Clinical Research Network (HCRN), published by Hauptmann et al. in 2020, showed a correlation between shunt‐revision rates and age at the time of the procedure, etiology of hydrocephalus, and the history of previous failure events. In 1030 patients, there were 1978 revision events. 38.5% failed within the first year after surgery [21, 22].

Ojo et al. quoted a shunt failure risk rate of around 40%, with a collective rate of 25.8%. With regard to shunt infection, the rate was 19.5%. In their work in 1998, Drake et al. described 8.1% of failures caused by infection. Other complications included shunt extrusion and dysfunction, as well as failure of the implant system or its components. Overall, endoscopic third ventriculostomies (ETVs) have a more favorable risk profile, with a risk of less than 10% [4, 6, 23, 24]. Multiloculated hydrocephalus arises from septations within the ventricular system. Endoscopic techniques have shown promise in restoring CSF circulation by facilitating the removal of obstructions and optimizing communication between compartments.

In addition to the traditional VP shunt, proven treatment concepts include microsurgical fenestration of cysts and placement of central ventricular or shunt catheters, particularly for multilocular hydrocephalus. Endoscopically assisted surgery has the obvious advantage of minimizing skull opening and brain retraction. Additionally, an endoscope facilitates deep access through preformed cavities, such as the ventricular system [2, 25, 26, 27].

VP‐catheters are often equipped with additional perforations to enable adequate drainage of the various compartments, and they are connected to each other via Y‐shaped connectors. Using a catheter has also been shown to be more effective than cyst fenestration alone [2, 11, 28]. Fibrinolytic reagents as well as consistent irrigation of the ventricular system seem to reduce the formation of septations somewhat [29].

In order to adequately counter the fundamental risk of colonization of a foreign body with germs [30], it is strongly recommended that, in addition to consistent infection treatment [3], nonfunctional implant remnants are removed.

Overall, this case highlights the importance of a multidisciplinary approach in providing optimal care for these vulnerable patients. Continued advancements in surgical techniques and infection prevention, alongside rigorous infection control, are vital for improving the quality of life and neurological outcomes for children with ICH, hydrocephalus, and recurrent infections.

## Author Contributions

**Hannes Egermann:** conceptualization, data curation, formal analysis, investigation, methodology, project administration, supervision, visualization, writing – original draft, writing – review and editing. **Basheer Al‐Shameri:** investigation, writing – review and editing. **Christian Knorr:** investigation, writing – review and editing. **Adolf Müller:** investigation, project administration, writing – review and editing. **Markus Denzinger:** investigation, project administration, supervision, visualization, writing – original draft, writing – review and editing. **Stephan Lackermair:** conceptualization, investigation, methodology, project administration, supervision, visualization, writing – original draft, writing – review and editing.

## Consent

Written informed consent was obtained before publication.

## Conflicts of Interest

The authors declare no conflicts of interest.

## Data Availability

The data supporting the findings of this case report are available from the corresponding author upon reasonable request. However, the raw data are not publicly shared in order to protect the privacy and confidentiality of the individual described in this report.
